# General Hospitals

**Published:** 1894-09-08

**Authors:** 


### The Institutional Workshop.

### HOSPITAL ADMINISTRATION.

(Third Series.)

### I.—GENERAL HOSPITALS.

In continuation of the series of articles which we have given in The Hospital during the past two years, we now place before our readers figures showing the percentage which the cost of administering our voluntary hospitals for the three years ended 1893 bore to the cost of maintenance, and also to the total ordinary expenditure. Until quite recently the difficulties in the way of making thorough comparisons between the relative success of various systems of administering medical institutions of the same size and importance were well-nigh insurmountable. Material was abundant, but each batch of facts had been compiled and put together upon the plan favoured by the compiler. Hence it proved impossible to form a definite and convincing judgment of the reasons which caused one hospital to expend half as much again, or twice as much again, per bed as another. Fortunately there was one factor at work which tended to build up the voluntary system—the friendly rivalry between the authorities as to which hospital could prove itself most worthy of public support. Still, the best administered hospitals were at a disadvantage in the absence of reliable data, compiled upon an identical basis, by which the public would be prevented from forming a wrong judgment as to relative merits. In such circumstances the prize of liberal offerings sometimes fell, as it often falls now, to the lot of institutions which are not among the best administered, although they may be the most successful in enlisting public sympathy by causing their work to be widely known.

Matters have, however, improved in many directions during the last few years. The adoption of the Uniform System of Accounts, and its enforcement by the Committee of Distribution of the Metropolitan Hospital Sunday Fund, is tending to remedy the old evils by making it more and more difficult for the relatively inefficient institutions to escape detection. The Council of the Metropolitan Hospital Sunday Fund has organised a system through its Committee of Distribution which enables any member of the public, by addressing a letter to the Lord Mayor at the Mansion House, to ascertain whether or no a particular hospital or medical charity is managed upon the most approved principles, and to what extent it worthily represents the highest form of administration at the time the inquiry is made. Hence there is no excuse for slipshod giving by wealthy donors and others who wish to wisely contribute of their abundance for the alleviation of sickness amongst the poor. The enforcement of a regular system of audit by public accountants, in conjunction with the governors' auditor, is also tending to promote efficiency of the highest kind.

#### The Expenses of Management.

The adoption of the uniform system of accounts has enabled reliable data to be prepared on an identical basis by the Metropolitan Hospital Sunday Fund, who have for some years past prepared tables showing the number of beds occupied each year; the number of home visits from dispensaries; the number of cases treated, with the approximate cost of treatment; the percentages of the cost of administration as compared with that of maintenance; the average amount of patients' payments; and the average gross annual income and expenditure during three years. For the past two years the Council have published in addition the percentage of the cost of administration to total expenditure. It is therefore possible to compare the working of each institution receiving a grant with every other institution on the list, and the results may be taken as a basis upon which thoughtful people may dispose of the amount of money they give each year to various hospitals.

We now proceed to examine what percentage the cost of administration is to (*a*) that of maintenance, and (*b*) to total expenditure, as shown in these returns.

The table on the next page contains the names of all the general hospitals which have 100 beds and upwards, in addition to the London Homœopathic Hospital. Speaking generally, in eight of the hospitals here given the percentage of the cost of administration to that of maintenance is higher in 1893 than in 1892, ranging from an increase of nearly 2½ per cent. at Guy's to $\frac{1}{25}$ of 1 per cent. at the German Hospital; while in six cases the percentage is lower, ranging from a decrease of $\frac{2}{5}$ of 1 per cent. to 4 per cent. In half the hospitals given the percentage of the cost of administration to the total expenditure is higher than in 1892.

A reference to pages 365 of The Hospital for August 27th, 1892, and 333 for August 19th, 1893, will enable the reader to follow in detail the progress or otherwise of each hospital included in the table. Roughly speaking, the percentage cost of administration to total expenditure has increased by nearly 3 per cent. at the Royal Free, nearly 2 per cent. at Guy's, nearly 1 per cent. at the Seamen's, and by under ½ per cent. at King's College, the West London, and St. Mary's, with a slight increase at the London. A reduction in cost of administration to total expenditure has taken place as follows: 3 per cent. at the London Homœopathic, 2 per cent. at University College and the Training Hospital (Tottenham), under 1 per cent. at St. George's and the Middlesex; and under ½ per cent. at Charing Cross, the Westminster, and the German.

When the percentage which the cost of administration bears to that of maintenance is considered, the results vary but little from those given above. Thus, compared with last year's returns, both University College and Guy's show an increase of over 2 per cent., King's and the Seamen's an increase of 1 per cent., and the Royal Free, West London, St. Mary's, the London, and the German under ½ per cent. increase. The remaining hospitals show a decrease of about 4 per cent. in the case of the London Homœopathic, of about 2 per cent. in the case of St. George's and the Training Hospital (Tottenham), and the Middlesex, Westminster, and Charing Cross Hospitals of under 1 per cent.

We may add, for the information of those who have not made a study of hospital finance, that a careful investigation of the whole of the questions involved, based upon information which reveals the circumstances of all the great hospitals of the world, has brought us to the conclusion that the expenditure, which it is essential that each committee shall sanction, in order to secure the maximum of efficiency in the administration of a voluntary general hospital, is represented by a maximum sum equal to 15 per cent. upon the total expenditure each year. This calculation relates to all general hospitals containing one hundred beds and upwards. Judged by this standard, it will be seen that none of the general hospitals in London exceed this limit, and that the actual percentage of the cost of administration to the total expenditure is now under 12 per cent. Bearing this in mind, and remembering the further fact that so many of the hospitals show a marked decrease in the percentage of cost during the year 1893 as compared with 1892, it may be fairly claimed that economy and efficiency are the watchwords of our great voluntary hospitals, and that the principles which they embody are steadily enforced by the managers is proved by the figures contained in the accompanying table.

Metropolitan General Hospitals.

*Table giving the averages for three years ended* 1893.

| Name of Hospital. | Beds in Occupation. | Average Number Occupied. | Total In-patients Admitted. | Total Out-patients Admitted. | Percentage of cost of Administration to that of Maintenance. | Percentage of Administration to Total Expenditure. |
|---|---|---|---|---|---|---|
| London ... ... ... | 776 | 656 | 9,945 | 127,094 | 5·356 | 4·816 |
| Guy's ... ... ... | 600 | 424 | 5,969 | 70,734 | 11·332 | 9·903 |
| St. George's ... ... | 351 | 323 | 4,201 | 34,657 | 3·233 | 3·132 |
| Middlesex ... ... | 321 | 215 | 2,513 | 30,252 | 5·697 | 5·390 |
| St. Mary's ... ... | 281 | 251 | 4,056 | 35,592 | 6·733 | 6·304 |
| Seamen's ... ... ... | 253 | 214 | 2,582 | 15,165 | 13·139 | 11·626 |
| King's ... ... ... | 220 | 185 | 2,372 | 23,308 | 12·067 | 10·311 |
| University ... ... | 207 | 186 | 3,228 | 49,486 | 11·481 | 10·399 |
| Westminster ... ... | 205 | 176 | 2,950 | 26,396 | 6·097 | 5·746 |
| Charing Cross ... | 175 | 150 | 2,503 | 23,001 | 11·520 | 10·339 |
| Royal Free ... ... | 160 | 112 | 1,554 | 25,131 | 7·578 | 7·044 |
| German ... ... ... | 120 | 104 | 1,175 | 22,071 | 12·709 | 11·167 |
| Training (Tottnhm.) | 103 | 64 | 962 | 8,516 | 3·987 | 3·643 |
| West London... ... | 101 | 97 | 1,519 | 31,446 | 11·780 | 10 538 |
| London Homœpthc. | 40 | 36 | 445 | 7,413 | 12·124 | 10·833 |

